# Investigating the Impact of Dragon Fruit Peel Waste on Starch Digestibility, Pasting, and Thermal Properties of Flours Used in Asia

**DOI:** 10.3390/foods11142031

**Published:** 2022-07-08

**Authors:** Siriwan Chumroenvidhayakul, Thavaree Thilavech, Mahinda Abeywardena, Sirichai Adisakwattana

**Affiliations:** 1 Phytochemical and Functional Food Research Unit for Clinical Nutrition, Department of Nutrition and Dietetics, Faculty of Allied Health Sciences, Chulalongkorn University, Bangkok 10330, Thailand; siriwan.chu112@gmail.com; 2 Department of Food Chemistry, Faculty of Pharmacy, Mahidol University, Bangkok 10400, Thailand; thavaree.thi@mahidol.ac.th; 3 CSIRO Health & Biosecurity, Kintore Avenue, Adelaide, SA 5000, Australia; mahinda.abeywadena@csiro.au

**Keywords:** dragon fruit peel, dietary fiber, in vitro starch digestion, physicochemical properties

## Abstract

As a by-product of dragon fruit consumption, dragon fruit peel (DFP) was developed into powder as a natural ingredient. Nevertheless, the effect of DFP on the physicochemical properties of flours used in Asian food processing and cooking remains unknown. In this study, starch digestibility, thermal, pasting, and physicochemical properties of DFP and flours (potato, rice, glutinous rice, and wheat) were characterized. It was found that DFP contained 65.2% dietary fiber together with phenolic compounds, betacyanins, and antioxidant activity. The results demonstrated that DFP (from 125 to 500 mg) reduced starch digestibility of flours, rapidly digestible starch, and slowly digestible starch, along with an increased proportion of undigested starch. A marked increase in phenolic compounds, betacyanins, and antioxidant activity occurred when DFP and flour were incubated for 180 min under simulated gastrointestinal digestion. The results indicate that bioactive compounds in DFP were highly bioaccessible and remained intact after digestion. Moreover, DFP exerted a significantly lower gelatinization enthalpy of flours with increasing peak viscosity and setback with decreasing pasting temperature. FTIR confirmed the decreased ratio at 1047/1022 cm^−1^, indicating the disruption of short-range orders of starch and DFP. These findings would expand the scope of DFP food applications and provide a knowledge basis for developing DFP flour-based products.

## 1. Introduction

Carbohydrates are the essential macronutrients that provide energy to the body, supporting functions and physical activity. Long-term carbohydrate overconsumption has been associated with increased blood glucose and visceral fat storage, promoting insulin resistance and inflammation, and raising the risk of metabolic syndrome, type 2 diabetes, and cardiovascular diseases (CVDs) [1,2]. Starch, a polysaccharide composed of glucose monomers, accounts for about 60% of the carbohydrates consumed by humans. In general, starches from wheat, rice, glutinous rice, and potatoes are the most popular ingredients used for culinary applications. Nevertheless, different types of flour provide unique physicochemical characteristics for food products and desserts, such as thickening, gelling, stabilizing, and moisture retention [3]. In addition to physicochemical properties, starches notably consist of different amylose to amylopectin ratios, affecting starch digestibility in the gastrointestinal tract [4]. In clinical studies, the consumption of high-amylose starch has been shown to reduce postprandial glycemic and insulinemic responses in healthy men and women compared to conventional starch [5,6]. Moreover, the reduced hydrolysis rate of starch delays carbohydrate digestibility, leading to the suppression of postprandial glycemic response [7].

Substituting or incorporating dietary fibers and phytochemical compounds into starches has recently been viewed as a practical approach for glycemic control by inhibiting the activity of carbohydrate digestive enzymes and interacting with their granules to modify swelling grade, gelatinization, and pasting properties [7,8]. For instance, apple peel containing 58.44% of dietary fibers effectively suppressed starch digestibility and decreased the glycemic index of wheat flour during simulated gastrointestinal digestion [9]. In addition, the incorporation of anthocyanin-rich extract from *Clitoria ternatea* flower into different flours such as potato, cassava, rice, corn, wheat, and glutinous rice resulted in a significant decrease in starch hydrolysis and glycemic index [10].

White pulp Dragon fruit (*Hylocereus undatus*), an economically important tropical fruit crop, is a low glycemic index food with a high concentration of vitamins, minerals, phytochemical compounds, and dietary fibers [11]. Furthermore, it has several biological properties, including antioxidant and anti-diabetic activity [12]. In Thailand, the pulp of white pulp dragon fruit is generally served on a patient menu in hospitals due to its nutritional benefits and inexpensive cost [13]. Unfortunately, it may contribute to environmental issues by creating vast amounts of peel waste, approximately 22% of the whole fruit [14]. To utilize this waste, dragon fruit peel (DFP) has become a potential ingredient in developing functional foods because it comprises soluble and insoluble fibers up to 69.3% of dry weight [14]. In recent decades, DFP has been used as an ingredient to improve the nutritional value of food products with high consumer acceptability and palatability, such as noodles, steamed bread, and cookies. [12]. Although DFP is a promising food ingredient for incorporation into various types of starch and flour, its effects on the physicochemical and functional properties of flour remain unclear. Therefore, it is of great significance to explore the interaction between DFP and flours used in Asian food processing and cooking. The objective of this study was to investigate the physicochemical and functional properties of DFP received from hospital food waste. Moreover, the effect of DFP on in vitro starch digestibility, pasting, and gelatinization with various types of flour (potato, glutinous rice, rice, and wheat flour) used in Asia was further explored. The research conclusions would provide new insights into the use of DFP in developing innovative flour-based functional products.

## 2. Materials and Methods

### 2.1. Materials

The dragon fruit peel was obtained from Ramathibodi Hospitals, Bangkok, Thailand. Commercial potato, glutinous rice, rice, and wheat flour were purchased from a supermarket. Folin-Ciocalteu reagent, betanin, 2,4,6-tripyridyl-s-triazine (TPTZ), pepsin from porcine gastric mucosa powder, α-amylase Type VI-B from porcine pancreas, and pancreatin from porcine pancreas were obtained from Sigma-Aldrich Chemical (St. Louis, MO, USA). Amyloglucosidase from *Aspergillus niger* was purchased from Roche Diagnostics (Indianapolis, IN, USA).

### 2.2. Dragon Fruit Peel Powder (DFP) Preparation

The fresh dragon fruit peel was cleaned to remove impurities and dried at 60 °C for 12 h using a hot air oven. Next, the dry material was milled (DXM-500, DX-FILL, Samut Prakan, Thailand) and sifted through No. 40 sieves to obtain the powder. Finally, DFP was stored in polyethylene foil bags and kept at −20 °C.

### 2.3. Proximate Analysis of DFP

The proximate composition of DFP, including moisture, ash, protein, total fat, total sugar, and total dietary fiber, was conducted according to the Association of Official Agricultural Chemists (AOAC) method (2016).

### 2.4. Determination of Total Phenolic Content (TPC), Total Betacyanin Content (TBC), and Antioxidant Activity

The TPC, TBC, and ferric reducing antioxidant power (FRAP) of DFP were determined according to previous studies [15,16]. The total phenolic content of DFP was determined by the Folin–Ciocalteu method. DFP solution (0.01 g/mL, 50 μL) was mixed with 50 μL of a 10-fold dilution of Folin–Ciocalteu’s reagent and incubated for 5 min. Then, 50 μL of 10% (*w/v*) Na_2_CO_3_ was added to the solution and further incubated for 60 min at room temperature. The absorbance was read at 750 nm. The results were expressed as mg of gallic acid equivalents (GAE) per gram of DFP powder.

Total betacyanin content in DFP powder was determined according to the previously described method by Tze et al. (2012) [17]. The DFP solution (10% *w/v* in distilled water) was sonicated for 1 h at room temperature. Then, the solution was centrifuged at 1904× *g* at 4 °C for 15 min. The supernatant was filtered through Whatman No. 1 filter paper. The sample solution was measured at 538 nm. The total betacyanin content was expressed as g of betanin equivalent per gram of DFP powder.

To make the fresh reagent for FRAP assay, 0.3 M acetate buffer (pH 3.6), 10 mM 2, 4, 6-tripyridyl-s-triazine (TPTZ) solution, 40 mM HCl, and 20 mM FeCl_3_ were mixed in the following proportions: 10:1:1. DFP solution (10% *w/v* in distilled water, 10 μL) was mixed with 90 μL of FRAP reagent and incubated for 30 min at room temperature. The absorbance was read at 595 nm. The FRAP value of DFP was expressed in mmol FeSO_4_ equivalent per gram of DFP powder.

### 2.5. Chemical Properties of DFP Pectin

The pectin content in DFP powder was extracted according to a previous study [17]. Five grams of DFP powder was mixed with 150 mL of distilled water and adjusted to pH 2.0. The solution was incubated at 75 °C for 1 h at 150 rpm. The solution was filtered and precipitated with an equal volume of 96% (*v/v*) ethanol for 1 h. Then, the precipitated pectin was dried at 60 °C for 5 h. The extracted DFP pectin was stored at room temperature for further analysis.

The equivalent weight (EW) of DFP pectin was calculated according to the equation below:

EW (g/mol) = weight of sample (g) × 1000/mL of alkali × Normality of alkali(1)

The methoxyl content was calculated according to the equation below:

MeO (%) = mL of alkali × Normality of alkali × 31 × 100/weight of sample(2)

where 31 is the methoxy group molecular weight.

The degree of esterification (DE) was determined by mixing DFP pectin powder (50 mg) with 10 mL of isopropanol (65%, *v/v*) and phenol red indicator. The solution was titrated with 0.1 N NaOH solution (A) to pink color. Then, a solution was added with 30 mL of 0.1 N NaOH and kept for 30 min, followed by 30 mL of 0.1 N HCl. The solution was further titrated with 0.1 N NaOH (B) to pH 7.5. DE was calculated by the following formula:

DE (%) = (B/A+B) × 100(3)

Total anhydrouronic acid (AUA) content in DFP pectin was calculated to determine the pectin purity by the following formula:

AUA (%) = ((176 × (0.1 × Z) × 100)/(W × 1000)) + ((176 × (0.1 × y) × 100)/(W × 1000))(4)

where molecular unit of AUA (1 U) = 176 g; Z = volume of NaOH from equivalent weight determination; y = volume of NaOH from methoxyl content determination; W = weight (g) of sample.

### 2.6. Physicochemical Properties of DFP

To determine the functional properties of DFP, oil holding capacity (OHC), water holding capacity (WHC), and swelling capacity (SC) were conducted following the previous study compared with sodium carboxymethyl cellulose (CMC) which is a commonly used food additive in the food industry [18].

#### 2.6.1. Oil Holding Capacity (OHC)

First, 250 mg of sample was mixed with 10 mL of rice bran oil. The solution was mixed for 1 min and left for 1 h at room temperature. Then, the sample was centrifuged at 1904× *g* for 5 min at room temperature. After removal of the supernatant, the sediment was weighed to calculate the OHC following the equation:

OHC (g of oil⁄g of sample) = weight of sediment (g) − weight of sample(g)(5)

#### 2.6.2. Water Holding Capacity (WHC)

Briefly, 250 mg of sample was placed in a centrifuge tube, and 10 mL of distilled water was added. The solution was mixed for 1 min and left for 1 h at room temperature. Then, the sample was centrifuged at 1904× *g* for 5 min. After removal of the supernatant, the sediment was weighed to calculate WHC using the following equation:

WHC (g of water⁄g of sample) = weight of sediment (g) − weight of sample (g)(6)

#### 2.6.3. Swelling Capacity (SC)

Two grams of DFP were weighed in a calibrated cylinder, and the initial volume (A) was recorded. Then, 100 mL of distilled water was added and left for 20 h at room temperature. The final volume (B) was calculated by using the following equation:

SC (mL of water⁄g of sample) = (A − B)/sample weight (g)(7)

### 2.7. In Vitro Starch Digestion and Starch Fractions

The impact of DFP with potato (P), glutinous rice (G), rice (R), or wheat (W) flour on in vitro starch digestion and total starch content was performed as described in the previous studies [19,20]. Prior to digestion, 500 mg of various types of flour, including potato flour, rice flour, glutinous rice flour, and wheat flour, were mixed with 5 mL of distilled water and heated for gelatinization. Then, 125, 250, and 500 mg of DFP were added to the gelatinized starch. The samples were then mixed with 1 mL of artificial saliva solution (250 U/mL porcine amylase in 0.2 M carbonate buffer, pH 7) and 5 mL of pepsin suspension (1 mg/mL) in 0.02 M HCl (pH 2). The mixtures were incubated for 1 h at 37 °C in a water bath shaker (100 rpm), followed by neutralization with 5 mL of 0.02 M NaOH and 25 mL of 0.2 M sodium acetate buffer (pH 6). The intestinal digestion was initiated by adding 5 mL of the enzyme mixtures (pancreatin (2 mg/mL) and amyloglucosidase (28 U/mL) to 0.2 M acetate buffer, pH 6. Next, the mixtures were further incubated at 37 °C, 100 rpm, and collected at 0, 20, 30, 60, 90, 120, and 180 min. The digesta fluid was immediately placed in an ice bath and centrifuged at 10,845× *g* for 15 min at 4 °C, then filtered through a 0.22 μm nylon filter to stop the reaction. The concentration of glucose was determined by Glucose LiquiColor^®^ (HUMAN GmbH, Wiesbaden, Germany). The percentage hydrolysis index (HI) was calculated by dividing the area under the hydrolysis curve of each sample by the corresponding area of standard glucose.

Total starch content was performed by mixing 50 mg of samples with 6 mL of 2 M KOH and incubating at room temperature for 1 h. Then, 3 mL of 0.4 M sodium acetate buffer was added, and the pH was adjusted to 4.75 before adding 60 µL of amyloglucosidase (3260 U/mL) and incubating for 45 min at 60 °C in a water bath shaker (100 rpm). The mixture was heated at 100 °C and further centrifuged at 18,327× *g*, for 5 min, at 4 °C. The concentration of glucose was determined and converted into the starch fraction by multiplying with 0.9. The total starch (TS) was calculated from the described equations:

RDS (%) = (G_20_ − G_0_)/TS × 100 (8)

SDS (%) = (G_120_ − G_20_)/TS × 100 (9)

Undigested starch (%) = (TS − (RDS + SDS))/TS × 100(10)

where G_0_ = glucose content after 0 min of digestion; G_20_ = glucose content after 20 min of digestion; G_120_ = glucose content after 120 min of digestion; TS = total starch content.

### 2.8. Determination of Total Phenolic Content (TPC), Total Betacyanin Content (TBC), and Antioxidant Activity during In Vitro Digestion

To investigate the impact of in vitro digestion on active compounds and antioxidant activity of DFP with potato (P), glutinous rice (G), rice (R), or wheat (W) flour. The digesta fluid at 0, 20, 30, 60, 90, 120, and 180 min was measured for total phenolic, betacyanin content, and FRAP according to the above-mentioned protocol.

### 2.9. Thermal, Pasting Properties and Fourier Transform Infrared (FT-IR) Spectroscopy

The thermal, pasting characteristics and FTIR spectra of each type of flour incorporated with DFP were investigated. The DFP–flour ratio at 250:500 was chosen for the experiments according to the significant reduction of starch digestibility.

#### 2.9.1. Thermal Properties

The gelatinization parameters, including onset temperature (T*o*), peak temperature (T*p*), the temperature at the conclusion (T*c*), and gelatinization enthalpy (ΔH), were recorded to investigate the impact of DFP on starch chains dissociating and the granules lost in all flours during gelatinization by differential scanning calorimetry (Netzsch DSC 204F1 Phoenix, Selb, Germany). Three milligrams (dry basis) of the sample were suspended with 10 µL of deionized distilled water and put into the sample pan and hermetically sealed. Then, it was allowed to stand for 1 h at room temperature for equilibration. The heating temperature was raised from 25 °C to 100 °C at a rate of 10 °C/min. An empty aluminum pan was used as the reference.

#### 2.9.2. Pasting Properties

The pasting profile of flours, including peak viscosity (PV), trough, breakdown (BD), final viscosity (FV), setback, and pasting temperature with or without 250 mg of DFP were evaluated by using a Rapid Visco Analyzer (RVA 4500, Newport Scientific Instrument, Newport, MN, USA). Three grams of the sample was dissolved in 25 mL of DW. The solution was heated at a rate of 12 °C per min from room temperature to 95 °C. After holding the sample at 95 °C for 2–3 min, it was cooled to 50 °C at a rate of 12 °C/min.

#### 2.9.3. Fourier Transform Infrared (FT-IR) Spectroscopy

To examine the effect of DFP on starch-ordered structure in all flours, the FT-IR spectra of samples were recorded using a Nicolet™ iS™50 spectrometer equipped with a Smart iTR™ diamond ATR concave tip (Thermo Fisher Scientific, Waltham, MA, USA) according to the previous study [21].

### 2.10. Statistical Analysis

The results were expressed as means ± standard error of mean (SEM) with three replicated determinations for each treatment group (*n* = 3). The area under the curves (AUCs) was calculated using the trapezoid rule. Data were analyzed by independent sample t-tests or one-way ANOVA, followed by Duncan’s post hoc test. The statistically significant difference was defined as *p* < 0.05 among treatments. Statistical analysis was performed using IBM SPSS version 22.0 (International Business Machines Corporation, Armonk, NY, USA).

## 3. Results and Discussion

### 3.1. Proximate Compositions, Bioactive Compounds, and Antioxidant Activity of DFP

In this study, DFP was mainly carbohydrates (70.85%), which consisted of 65.17% dietary fibers and 5.68% of available carbohydrates. The other components were followed by 15.91% ash, 6.37% protein, and 5.81% moisture, while total fat was the lowest component (1.06%). TPC and TBC in DFP were 454.79 ± 18.72 mg of gallic acid equivalent/g powder and 335.34 ± 2.26 mg of betanin equivalent/g powder, respectively. In terms of antioxidant activity, the FRAP value of DFP was 49.30 ± 0.10 mmol FeSO_4_ equivalent/g powder. The findings, particularly dietary fibers, were lowered, while TPC and TBC were higher than those reported in red dragon fruit peel because of the species, cultivation, maturity, and preparation method [14,15]. Previous research indicates that cellulose and lignin were major fractions of insoluble dietary fiber in dragon fruit peel, while pectin and mucilage were also detected as soluble dietary fiber [14,22]. Furthermore, 17 types of betacyanins, mainly betanin, gallic acid, chlorogenic acid, syringic acid, and ferulic acid, were phytochemical compounds obtained from DFP, which exhibited strong free radical scavenging capacities toward FRAP, ABTS, and DPPH [12].

### 3.2. Pectin and Physicochemical Properties of DFP

The yield of pectin from the extraction was 10.37% of DFP. As shown in Table 1, the average EW of DFP pectin was 397.63 ± 1.51 g/mol, relating to the gel strength of pectin. DFP pectin contained 5.22 ± 0.11% MeO and 46.90 ± 3.03% DE, classified as low methoxyl pectin (DE ≤ 50%), whereas the total AUA content of DFP pectin was 79.45 ± 0.53%. Interestingly, DFP has a lower EW pectin level and a more significant proportion of apple peel than MeO, DE, and AUA [23]. According to the Food Chemicals Codex (2016), DFP may be advantageous for food applications because it contains a high purity of pectin, which can form a gel in the absence of sugar [18,24]. Regarding physicochemical properties, DFP had 1.67-fold and 1.74-fold higher WHC, and SC values compared to carboxymethyl cellulose (CMC), respectively. It suggests that the higher hydration properties of DFP may be attributed to the presence of insoluble fibers, which improve the syneresis, texture, and viscosity of food products [25]. However, there were no significant differences in OHC levels between DFP and CMC.

### 3.3. Effect of DFP on In Vitro Starch Digestion, Hydrolysis Index, and Starch Fraction

**Figure 1:**
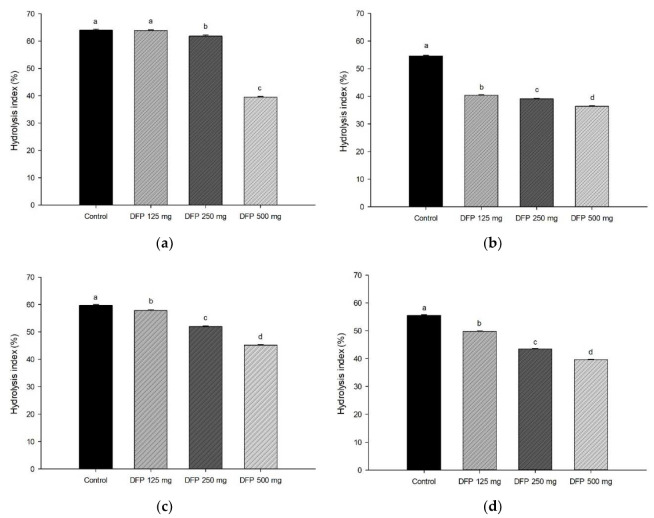
The effect of DFP addition on the percentage of hydrolysis index of (**a**) potato flour, (**b**) glutinous rice flour, (**c**) rice flour, and (**d**) wheat flour in combination with the different concentrations of DFP (125, 250, and 500 mg). The results are expressed as mean ± SEM, *n* = 3. Means with different superscripts are significantly different (*p* < 0.05).

**Figure 2:**
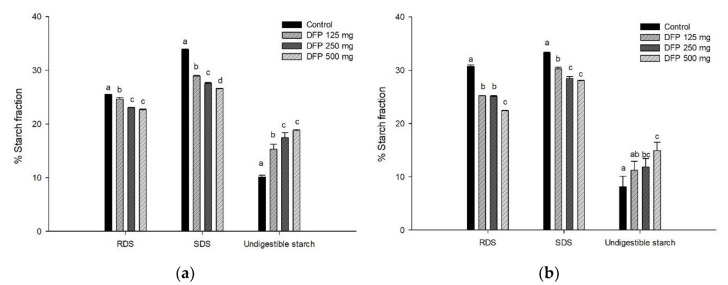

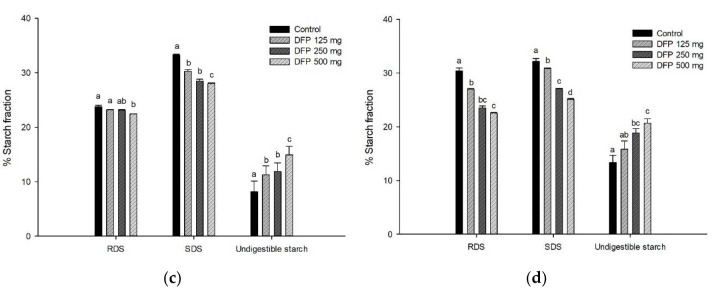
The effect of DFP addition on the percentage of starch fraction of (**a**) potato flour, (**b**) glutinous rice flour, (**c**) rice flour, and (**d**) wheat flour in combination with the different concentrations of DFP (125, 250, and 500 mg). The results are expressed as mean ± SEM, *n* = 3. Means with different superscripts are significantly different (*p* < 0.05). RDS—rapidly digestible starch; SDS—slowly digestible starch.

The amount of glucose released from all types of flour at different concentrations of DFP was reported as the area under the curves (AUC) during simulated gastrointestinal digestion (Table 2). The results showed that the AUC glucose of the mixture of DFP and each type of flour was lower than that of the control, indicating that the addition of DFP (500 mg) remarkably suppressed the starch digestibility. The greatest suppressing effect of DFP was observed when incorporated with potato flour (38.5%), followed by glutinous rice flour (33.5%), rice flour (24.4%), and wheat flour (25.5%). In addition, the percentage of hydrolysis index (HI) of potato, rice, glutinous rice, and wheat flour significantly decreased when incorporated with DFP (125–500 mg) in a concentration-dependent manner (Figure 1). According to the proximate analysis, dietary fibers in DFP, a major component, may play a role in interfering with the starch digestion process through trapping starch granules, increasing viscosity, and diminishing enzyme accessibility [26]. Moreover, betacyanins in DFP, especially betanin, could inhibit carbohydrate digestive enzymes, including α-amylase and α-glucosidase, as supported by a previous study of betacyanin-rich beetroot juice [27]. The interactions of phenolic compounds with starch granules cause the formation of amylose single helices or complexes through hydrogen bonds, leading to a reduction in starch digestibility and HI [28]. Bae et al. reported that the incorporation of apple peel dietary fibers (mainly insoluble dietary fibers) into wheat flour decreased the starch hydrolysis, corresponding to the reduction of RDS and SDS, whereas the undigestible starch content increased [9]. These results are consistent with our findings that the addition of DFP (500 mg) significantly decreased RDS (3.1–13.9%) and SDS (6.1–13.6%) of all types of flour (Figure 2). The percentage of undigested starch increased in the flour added with DFP (500 mg) by 20.6% for potato flour, 8.9% for glutinous rice flour, 21.2% for rice flour, and 5.2% for wheat flour. From this nutritional point of view, the decrease in RDS and SDS, concomitant with the increase in undigested starch, is considered an effective way to control postprandial glycemic and insulinemic responses, leading to a lower risk of chronic degenerative illnesses such as type 2 diabetes and obesity [29].

### 3.4. Effect of DFP on TPC, TBC, and Antioxidant Activity during In Vitro Digestion

**Figure 3:**
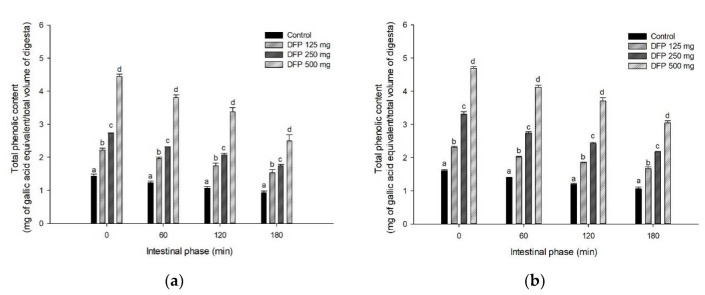

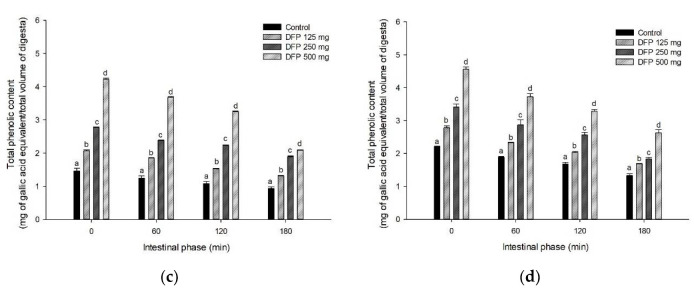
The release of total phenolic content (TPC) of (**a**) potato flour, (**b**) glutinous rice flour, (**c**) rice flour, and (**d**) wheat flour in combination with the different concentrations of DFP (125, 250, and 500 mg) during in vitro digestion. The results are expressed as mean ± SEM, *n* = 3. Means with different superscripts are significantly different (*p* < 0.05).

**Figure 4:**
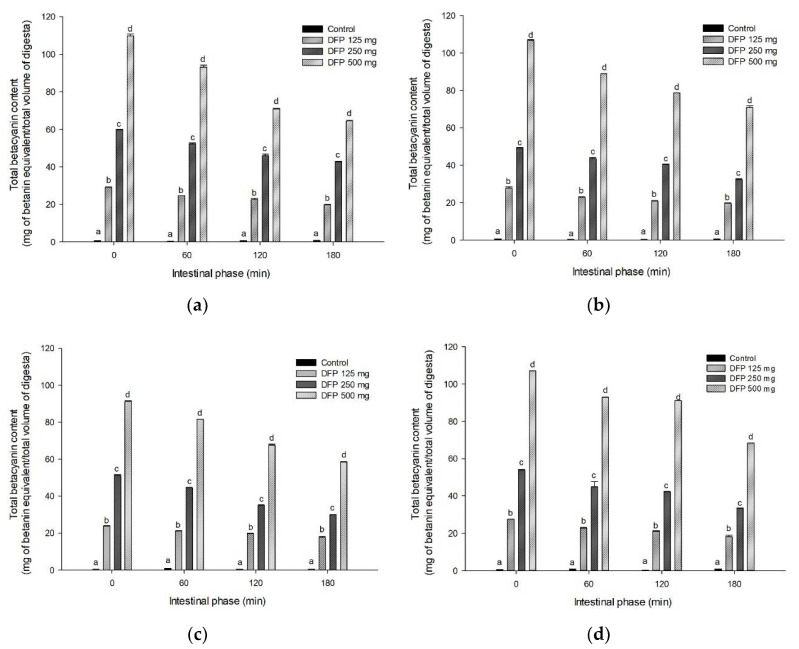
The release of total betacyanin content (TBC) of (**a**) potato flour, (**b**) glutinous rice flour, (**c**) rice flour, and (**d**) wheat flour in combination with the different concentrations of DFP (125, 250, and 500 mg) during in vitro digestion. The results are expressed as mean ± SEM, *n* = 3. Means with different superscripts are significantly different (*p* < 0.05).

**Figure 5:**
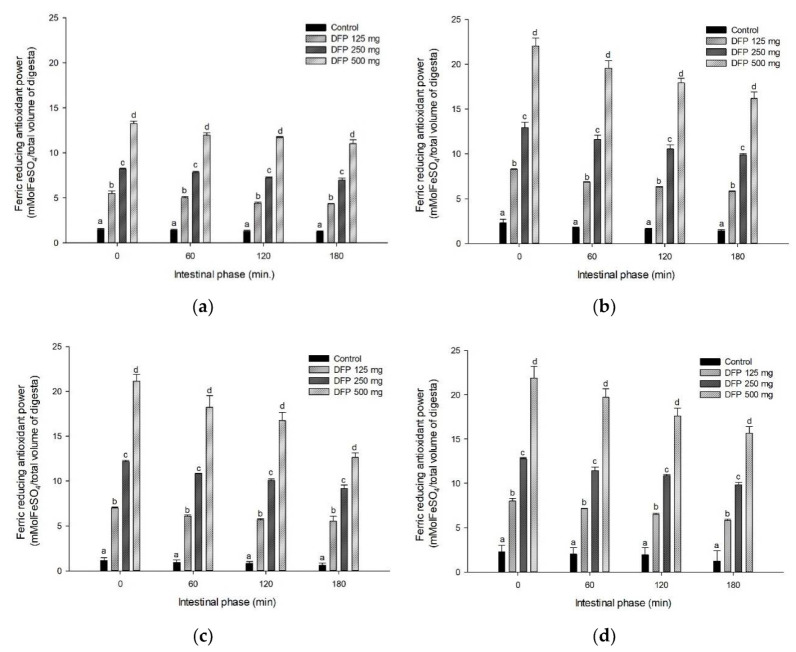
The ferric reducing antioxidant power (FRAP) of (**a**) potato flour, (**b**) glutinous rice flour, (**c**) rice flour, and (**d**) wheat flour in combination with the different concentrations of DFP (125, 250, and 500 mg) during in vitro digestion. The results are expressed as mean ± SEM, *n* = 3. Means with different superscripts are significantly different (*p* < 0.05).

The effects of DFP on TPC, TBC, and antioxidant activity of flours are shown in Figure 3, Figure 4 and Figure 5. The results showed that TPC, TBC, and antioxidant activity (FRAP) were detected in all flours and remained unchanged in intestinal digestion (0–180 min). Interestingly, DFP exhibited a significant increase in antioxidant activity and the release of TPC and TBC when added to flour. As presented in Table 2, the addition of DFP (125–500 mg) to different flours enhanced the increase in the AUC_TPC_ and AUC_TBC_ during simulated digestion in a concentration-dependent manner. Consistent with TPC and TBC, the antioxidant activity of flours presented by the AUC_FRAP_ was significantly higher in the presence of DFP when compared to the flour control.

In general, the food matrix containing various types of starch significantly influences the bioaccessibility and bioavailability of bioactive compounds and their biological activity. Our results indicate that bioactive compounds (total phenolic compounds and betacyanins) in DFP are highly bioaccessible and remained intact after simulated starch digestion, contributing to the antioxidant activity. This finding is consistent with the previous study indicating that betacyanins from red dragon fruit were retained after simulated gastrointestinal digestion and demonstrated their antioxidant activity [30]. Next, we investigated the correlation between the effects of DFP on the reduction of glucose released from flour, TPC, TBC, and antioxidant activity after simulated digestion (as shown in Figure 3, Figure 4 and Figure 5). As shown in Table 3, the AUC_Glucose_ showed a significantly negative correlation with the AUC_TPC_, AUC_TBC_, and AUC_FRAP_ (r = −0.786, −0.688, and −0.755, respectively). Interestingly, the AUC_TPC_ and AUC_TBC_ were significantly positively correlated with the AUC_FRAP_. These results support the notion that phenolic compounds and betanin released during in vitro simulated digestion may play a vital role in antioxidant activity and suppressing starch digestibility [28,31].

### 3.5. Effect of DFP and Various Types of Flour on Thermal Properties

The thermal parameters of DFP (250 mg) with different flours are summarized in Table 4. The onset temperature (T*_o_*) and peak temperature (T*_p_*) of potato, glutinous rice, and wheat flour mixed with DFP were significantly increased, except for rice flour. Furthermore, the conclusion temperature (T*_c_*) of flours mixed with DFP also increased significantly. The gelatinization enthalpy (ΔH) of potato, glutinous rice, rice, and wheat flour was lowered by 2.38, 1.19, 2.22, and 1.88 times, respectively, when DFP was added. The gelatinization enthalpy is an essential thermal energy that causes the swelling, crystallite melting, and solubilization of starch granules [32]. Soluble dietary fibers have been shown to reduce gelatinization enthalpy, resulting in the limitation of the susceptibility of starch digestibility [33]. In this study, DFP demonstrates a high-water holding capacity which competes for available water with starch granules and disrupts the melting crystalline structures of starch granules. Consequently, this effect causes interference with starch granule swelling and increases the amount of ungelatinized starch [34].

The pasting properties of flour incorporated with DFP are shown in Table 5. The pasting properties of potato, glutinous rice, rice, and wheat flour were significantly altered by adding DFP (250 mg). Moreover, DFP markedly increased the peak viscosity (PV), trough viscosity, final viscosity (FV), and breakdown viscosity (BD) of flours.

In agreement with a previous study, the high hydration property of DFP may compete to attach water with starch granules, causing the rupture of starch granules and hindering the release of amylopectin, which is linked to interfering with the formation of starch gel and chain network [35]. In addition, the setback viscosity of the flour incorporated with DFP was higher than that of the control flour. The results indicate that DFP alters starch’s solubility, gel formation, and retrogradation, potentially reducing its structure and digestibility [32].

Moreover, the pasting temperature of all flours was significantly decreased after DFP incorporation. These results suggest that dietary fibers in DFP interfere with flour’s gelatinization and heat stability [36].

**Figure 6:**
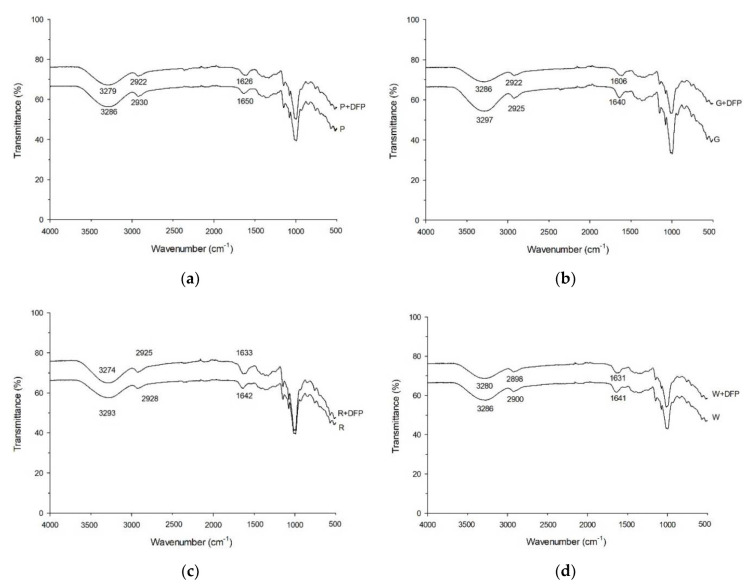
Fourier transform infrared spectrometer (FT-IR) spectra of (**a**) potato flour, (**b**) glutinous rice flour, (**c**) rice flour, and (**d**) wheat flour in combination with 250 mg the dragon fruit peel powder (DFP).

The interaction between various types of flour and DFP observed by the Fourier transform infrared spectrometer (FT-IR) is displayed in Figure 6. The essential characteristic peaks of flour were characterized in this study, including stretching vibrations of the O-H bond (3200–3300 cm^−1^), the C-H bond (2900–3000 cm^−1^), the C=O bond, and water absorption in the amorphous region (1640 cm^−1^). Following the addition of DFP (250 mg), the characteristic peaks in all flours were shifted to lower wavenumbers, indicating the interaction between starch and DFP. These interactions were also observed when mixing phenolic compounds, proanthocyanidins, and soluble dietary fiber with flour, causing a reduction in starch digestibility [21,37]. In general, the ratio of the bands at 1047, 1022, and 1000 cm^−1^ represents crystalline and amorphous structures in starch, which refer to amylose and amylopectin content and affect the starch digestibility. For example, higher amylose content and a lower proportion of amylopectin in rice starches have been associated with reduced susceptibility to enzymatic hydrolysis, providing higher resistance to enzymatic digestion [38,39]. In the present study (Table 5), adding DFP into flour decreased the ratio of 1047/1022 cm^−1^ while increasing the ratio of 1022/1000 cm^−1^. The results suggest that phytochemical compounds and dietary fibers in DFP may interfere with the melting of the crystalline region by strengthening the amylose chain (amorphous part) and reducing hydrogen bonds, leading to slow digestibility flours [40,41]. However, further study is needed to clarify the impact of DFP on amylose content in all flours.

## 4. Conclusions

DFP powder made from the by-product of white pulp dragon fruit consumption contains dietary fibers together with phytochemical compounds and antioxidant activity. DFP significantly decreased starch digestibility of flour with an increase in undigested starch and higher antioxidant activity. In addition, it altered flours’ gelatinization enthalpy and pasting properties through interference with crystalline and amorphous structures in starch. These findings would expand the scope of DFP applications and provide a knowledge base of DFP waste for developing flour-based products.

## Figures and Tables

**Table 1:** Pectin chemical property and physicochemical property of dragon fruit peel powder (DFP).

	Pectin Chemical Property				Physicochemical Property		
	**EW** **(g/mol)**	**MeO** **(%)**	**DE** **(%)**	**AUA** **(%)**	**OHC** **(g of Oil/g of Sample)**	**WHC** **(g of Water/g of Sample)**	**SC** **(mL of Water/g of Sample)**
CMC	N.A.	N.A.	N.A.	N.A.	1.63 ± 0.06 ^a^	5.64 ± 0.16 ^a^	2.63 ± 0.06 ^a^
DFP	397.63 ± 1.51	5.22 ± 0.11	46.90 ± 3.03	79.45 ± 0.53	1.93 ± 0.03 ^a^	9.44 ± 0.19 ^b^	4.60 ± 0.03 ^b^

Data are expressed as mean ± SEM, *n* = 3. Means with different superscripts are significantly different (*p* < 0.05). EW—equivalent weight; MeO—methoxyl content; DE—degree of esterification; AUA—total anhydrouronic acid; OHC—oil holding capacity; WHC—water holding capacity; SC—swelling capacity; CMC—sodium carboxymethyl cellulose; N.A.—not analyzed.

**Table 2:** The effect of the addition of dragon fruit peel powder at 125 (DFP125), 250 (DFP250), and 500 (DFP500) mg on the area under the curve (AUC) of released glucose (AUC_glucose_), total phenolic content (AUC_TPC_), total betanin content (AUC_TBC_), and ferric reducing antioxidant power (AUC_FRAP_) of 500 mg of potato flour (P), glutinous rice flour (G), rice flour (R), and wheat flour (W) during in vitro starch digestion.

Experiments	AUC_Glucose_	AUC_TPC_	AUC_TBC_	AUC_FRAP_
P	8900.6 ± 82.5 ^a^	208.1 ± 9.0 ^a^	N.D.	241.4 ± 13.8 ^a^
P + DFP125	8815.9 ± 46.6 ^a^	334 ± 10.1 ^b^	4250.3 ± 28.8 ^a^	858.8 ± 14.6 ^b^
P + DFP250	8520.9 ± 62.6 ^b^	394.9 ± 4.2 ^c^	8878.2 ± 28.7 ^b^	1350.0 ± 19.1 ^c^
P + DFP500	5474.2 ± 34.0 ^c^	643.6 ± 16.5 ^d^	14,545.4 ± 32.6 ^c^	2146.2 ± 20.8 ^d^
G	7551.1 ± 111.5 ^a^	236.3 ± 3.2 ^a^	N.D.	307.4 ± 15.3 ^a^
G + DFP125	5597.8 ± 88.7 ^b^	349.6 ± 2.5 ^b^	4045.1 ± 6.6 ^a^	1199.4 ± 50.7 ^b^
G + DFP250	5405.5 ± 78.6 ^b^	466.1 ± 1.6 ^c^	7464.8 ± 18.9 ^b^	1990.9 ± 50.4 ^c^
G + DFP500	5020.2 ± 78.0 ^c^	699.6 ± 4.1 ^d^	15,363.8 ± 16.3 ^c^	3447.7 ± 29.5 ^d^
R	8261.6 ± 27.3 ^a^	209.6 ± 10.0 ^a^	N.D.	155.7 ± 27.5 ^a^
R + DFP125	7989.2 ± 47.1 ^b^	303.7 ± 2.3 ^b^	3705.3 ± 20.8 ^a^	1069.5 ± 11.1 ^b^
R + DFP250	7179.2 ± 46.8 ^c^	414.8 ± 3.2 ^c^	6984 ± 10.0 ^b^	1887.2 ± 20.7 ^c^
R + DFP500	6244.2 ± 15.0 ^d^	606.4 ± 4.6 ^d^	13,490 ± 26.7 ^c^	3270.9 ± 48.1 ^d^
W	7684.8 ± 42.8 ^a^	320.6 ± 2.7 ^a^	N.D.	370.0 ± 41.8 ^a^
W + DFP125	6884.4 ± 28.1 ^b^	392.3 ± 2.5 ^b^	4011.6 ± 15.0 ^a^	1218.4 ± 14.3 ^b^
W + DFP250	6001.2 ± 19.0 ^c^	481.4 ± 6.0 ^c^	8024.5 ± 13.5 ^b^	2007.7 ± 18.1 ^c^
W + DFP500	5478.8 ± 87.2 ^d^	632.7 ± 5.7 ^d^	15,818.4 ± 7.4 ^c^	3217.8 ± 45.7 ^d^

The results are expressed as mean ± SEM, *n* = 3. Means with different superscripts are significantly different (*p* < 0.05). AUC_glucose_—g of glucose/100 g of sample×min; AUC_TPC_—mg of gallic acid equivalent/total volume of digesta×min; AUC_TBC_—mg of betanin equivalent/total volume of digesta × min; AUC_FRAP_—mmol FeSO_4_/total volume of digesta×min; N.D.—not detected.

**Table 3:** Pearson correlation coefficients between dragon fruit peel powder (DFP) and the area under the curve (AUC) of released glucose (AUC_Glucose_), total phenolic content (AUC_TPC_), total betanin content (AUC_TBC_), and ferric reducing antioxidant power (AUC_FRAP_) during in vitro starch digestion.

	AUC_Glucose_	AUC_TPC_	AUC_TBC_	AUC_FRAP_
AUC_Glucose_	1	−0.786 *	−0.688 *	−0.755 *
AUC_TPC_		1	0.965 *	0.948 *
AUC_TBC_			1	0.948 *
AUC_FRAP_				1

* Significant correlation at *p* < 0.01. Correlations were done using the average value for each treatment, *n* = 3, in a mixture of various types of flour (potato, glutinous rice, rice, and wheat flour).

**Table 4:** The effect of dragon fruit peel powder (DFP) on thermal properties of potato flour (P), glutinous rice flour (G), rice flour (R), and wheat flour (W).

Experiments	Thermal Properties			
	T*_o_* (°C)	T*_p_* (°C)	T*_c_* (°C)	ΔH (J/g)
P	64.85 ± 0.35	71.75 ± 0.55	79.95 ± 0.45	11.81 ± 0.10
P + DFP250	68.90 ± 0.10 *	76.75 ± 0.15 *	83.65 ± 0.65 *	4.96 ± 0.13 *
G	63.50 ± 1.30	69.10 ± 0.40	74.15 ± 0.45	5.47 ± 1.52
G + DFP250	66.80 ± 0.40 *	74.50 ± 0.10 *	79.30 ± 0.50 *	4.60 ± 1.58
R	67.15 ± 2.95	74.75 ± 2.35	82.05 ± 0.25	8.02 ± 0.17
R + DFP250	66.70 ± 0.00	72.50 ± 0.50	86.10 ± 0.50 *	3.61 ± 0.96 *
W	57.35 ± 0.25	63.70 ± 0.30	69.60 ± 0.30	5.93 ± 0.18
W + DFP250	61.55 ± 0.05 *	68.00 ± 1.00 *	72.50 ± 1.40 *	3.15 ± 0.27 *

The results are expressed as mean ± SEM, *n* = 3. * *p* < 0.05 when compared to the control flour without DFP. T*_o_*—onset temperature; T*_p_*—peak temperature; T*_c_*—conclusion temperature; ΔH—enthalpy gelatinization.

**Table 5:** The effect of dragon fruit peel powder (DFP) on pasting properties and FTIR ratio of potato flour (P), glutinous rice flour (G), rice flour (R), and wheat flour (W).

Experiments	Thermal Properties				FTIR Ratio			
	PV (RVU)	Trough (RVU)	BD (RVU)	FV (RVU)	Setback (RVU)	PT (°C)	1047/1022 cm^−1^	1022/1000 cm^−1^
P	267.08 ± 1.25	87.20 ± 0.13	179.88 ± 1.13	128.71 ± 0.04	41.50 ± 0.08	71.83 ± 0.08	1.107	1.024
P + DFP250	573.17 ± 1.42 *	287.42 ± 1.50 *	285.75 ± 0.08 *	395.79 ± 4.04 *	108.38 ± 5.55 *	70.50 ± 0.40 *	1.103	1.042
G	387.13 ± 2.83	254.75 ± 1.58	132.38 ± 0.79	318.88 ± 1.05	64.13 ± 0.55	71.78 ± 0.02	1.151	0.890
G + DFP250	521.77 ± 0.01 *	403.96 ± 2.21 *	117.79 ± 2.21 *	584.25 ± 2.17 *	180.30 ± 4.38 *	66.48 ± 0.48 *	1.084	1.174
R	156.13 ± 0.21	149.21 ± 0.13	6.92 ± 0.09	259.25 ± 0.58	110.04 ± 0.46	90.05 ± 0.35	1.167	0.905
R + DFP250	456.09 ± 2.84 *	433.08 ± 3.50 *	23.00 ± 0.67 *	604.25 ± 1.67 *	171.17 ± 1.83 *	70.53 ± 0.38 *	1.122	1.159
W	108.25 ± 0.92	66.63 ± 0.70	41.63 ± 0.20	151.88 ± 0.21	85.25 ± 0.92	88.83 ± 0.02	1.140	1.005
W + DFP250	456.50 ± 1.00 *	373.42 ± 2.34 *	83.09 ± 1.34 *	553.96 ± 2.04 *	180.54 ± 0.29 *	58.85 ± 0.45 *	1.078	1.044

The results are expressed as mean ± SEM, *n* = 3. * *p* < 0.05 when compared to plain flour. PV—peak viscosity; BD—breakdown value; FV—final viscosity; PT—pasting temperature; RVU—rapid visco unit.

## Data Availability

Data is contained within the article.
